# Spatial Analysis on Hepatitis C Virus Infection in Mainland China: From 2005 to 2011

**DOI:** 10.1371/journal.pone.0110861

**Published:** 2014-10-30

**Authors:** Lu Wang, Jiannan Xing, Fangfang Chen, Ruixue Yan, Lin Ge, Qianqian Qin, Liyan Wang, Zhengwei Ding, Wei Guo, Ning Wang

**Affiliations:** 1 National Center for AIDS/STD Control and Prevention, Chinese Center for Disease Control and Prevention, Beijing, China; 2 Beijing Human Resources and Social Security Bureau, Beijing, China; Temple University School of Medicine, United States of America

## Abstract

**Background:**

The burden of Hepatitis C virus (HCV) has become more and more considerable in China. A macroscopic spatial analysis of HCV infection that can provide scientific information for further intervention and disease control is lacking.

**Methods:**

All geo-referenced HCV cases that had been recorded by the China Information System for Disease Control and Prevention (CISDCP) during 2005–2011 were included in the study. In order to learn about the changes of demographic characteristics and geographic distribution, trend test and spatial analysis were conducted to reflect the changing pattern of HCV infection.

**Results:**

Over 770,000 identified HCV infection cases had specific geographic information during the study period (2005–2011). Ratios of gender (Male/Female, Z-value  = −18.53, P<0.001), age group (≤30 years old/≥31 years old, Z-value  = −51.03, P<0.001) and diagnosis type (Clinical diagnosis/Laboratory diagnosis, Z-value  = −130.47, P<0.001) declined. HCV infection was not distributed randomly. Provinces Henan, Guangdong, Guangxi, Xinjiang, and Jilin reported more than 40,000 HCV infections during 2005 to 2011, accounting for 43.91% of all cases. The strength of cluster of disease was increasing in China during the study period. Overall, 11 provinces had once been detected as hotspots during 7 years, most of which were located in the central or border parts of China. Tibet, Qinghai, Jiangxi were the regions that had coldspots.

**Conclusions:**

The number of clustering of HCV infection among older adults increased in recent years. Specific interventions and prevention programs targeting at main HCV epidemic areas are urgently in need in mainland China.

## Introduction

Hepatitis C virus (HCV) is known as the major cause of chronic liver disease, human hepatic cirrhosis, and even hepatocellular carcinoma [1]. The fact that more and more HCV infections or patients who living with HCV has become a severe public health problem [2]. WHO has estimated that there are more than 150 million people with chronic liver disease which were caused by HCV infection in 2012, and over 350,000 people has been dead from HCV-related diseases worldwide each year [3]. Due to lack of vaccine and unavailability of effective therapy, the prevention of HCV infection has been a great challenge, especially in China, which is the largest developing country and owns one-fifths of the world's population. The population of China had approached 1.35 billion at the end of 2011 [4]. Although the prevalence of HCV infection had been lower in China after mid-1990s and the overall prevalence of anti-HCV was less than 1% in recent years [5], [6], the huge size of China population makes the absolute number of people infected with HCV enormous.

HCV infection is a public issue, which has an association with the interrelated social, geographic, historical and economic elements, just like other infectious diseases. Although various studies have already shown the prevalence of HCV infection varies with the different geographic positions in China [1], [4], [7], most of existing studies mainly focused on the spatial distribution of HCV genotypes and limited researches on the spatial distribution of HCV infection itself [8]–[10]. Shunquan Wu et al used spatial technique to work on the prevalence of HCV infection in Fujian province, and found that the HCV infection did have a relationship with spatial factors and the cluster of disease [11]. Apart from this study, the HCV infections in some provinces were more serious than other surrounding provinces, which might attribute to poor economy, inadequate primary health infrastructure, or inappropriate treatments [1]. In addition, HCV infection shares the same transmission routes with HIV infection, which had been already observed cluster of disease in studies [12], [13]. Especially for the intravenous drug users (IDUs), the prevalence of co-infection of HIV and HCV are high [14], and the HIV epidemics have association with geographic factors [15].

Since there is a geographic indicator related to the HIV infection, a macroscopic spatial analysis of HCV infection in this country is needed to probe whether the distribution of HCV infection was randomly distributed or not, which might facilitate prevention and control for HCV epidemic. In this study, we utilize the data of HCV reported cases in mainland of China from the year of 2005 to 2011 to explore the potential relationship between HCV infection and geographic distribution by using geographical information systems (GIS) and spatial statistics, which including general spatial autocorrelation (a tool was used to measure and analyze the degree of dependency among observations in a geographic space) and local spatial autocorrelation (a tool was used to detect cluster of high-value or low-value of observations).

## Methods

### Data collection and management

In 2004, after the Severe Acute Respiratory Syndrome (SARS) outbreak, China has established a world's largest web-based disease reporting system, called China Information System for Disease Control and Prevention (CISDCP). Since then, all medical institutions should report diagnosed HCV infection cases to specific local Center for Disease Control and Prevention (CDC) through CISDCP. Demographic information (age, gender, address, registered residency places, time of onset of the disease etc.) were collected using standardized case report forms (CRFs) by conducting private interviews. After being checked, confirmed case reports were saved in the web-based system. The procedure of reporting HCV case through CISDCP is consistent around the whole country. This comprehensive and well-organized system, CISDCP, can provide both the number of HCV infection cases and the spatial distribution information of these cases across the country.

All HCV infection cases, identified during 2005 to 2011 in CISDCP, were included. In order to identify the residential locations of the reported HCV infection cases, the corresponding national standard geocodes at city level were included in the analysis. Any personal identifiers which might reveal the privacy of the participants were removed before data analysis in this study. Electronic maps were obtained from China CDC (CCDC). ArcGIS 10.1 software (ESRI Inc., Redlands, CA, USA) was used to create electronic maps and SPSS18.0 software (IBM Inc., Armonk, NY, USA) was used to process and analyze the data.

The data in this study was based on HCV regular monitoring system in China, and related ethics committee and Chinese government have approved for the system to collect patient data. Since this study focused on population-level analyses only and did not access any individually identifiable patient data, ethics committee approval was not particularly required in this study.

Anyone can apply for using the data in ***The data-center of China public health science*** (http://www.phsciencedata.cn/Share/en/data.jsp?id=9906073c-200a-4b44-8ffe-0867bfa42557) or email to data@chinacic.cn.

### Trend analysis

We used Cochran-Armitage trend test to analyze the changing patterns of demographic and other characteristics, by ratios of gender (male/female), age group (≤30 years old/≥31 years old) and diagnosis type (clinical diagnosis/laboratory diagnosis) of the identified HCV infection cases from 2005 to 2011. In the present study, α = 0.05 has been selected as the level of significance for the test.

### General spatial autocorrelation

General spatial autocorrelation test statistic is a technique which is able to measure and analyze the spatial clusters in the data, and calculate the degree of dependency among observations in the whole geographic space [16]. In our study, general Moran's Index was used to discover and measure the HCV infection clusters in mainland China. The value of Moran's Index was set between [−1, 1]. When the value of general Moran's Index was >0 and Z-value >1.96, or the general Moran's Index was <0 and Z-value <−1.96, it indicated that the distribution of identified HCV infection cases clustered in the whole area; otherwise, the distribution of the infection cases was random. The specific formula of general Moran's Index was calculated as: [12]:
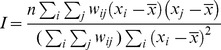



Where n is the number of spatial units (cities), x_i_ and x_j_ were the observations from unit i to unit j about the phenomenon X; w_ij_ represents the adjacent weight matrix. If the unit i was adjacent to the unit j, then w_ij_ would be 1; otherwise, it would be 0. In order to avoid the human influence of distance band of matrix, the threshold distance between cities was used in this study.

### Local spatial autocorrelation

This method, also name local indicator of spatial association (LISA), was initially created to detect the clustering of cases of rare diseases [17]. The method focused on detecting specific local clusters of cases without any preconception about their locations. In other words, the aim of local spatial autocorrelation is to recognize clusters which may not be identifiable by general spatial autocorrelation. The Getis statistics was chosen as the parameter to identify the local clusters in the present study, and the formula of it was showed as below [12]:
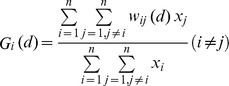



The meaning of parameters are similar as general Moran's Index formula, Z-test was conducted for the Gi parameter. If Z-value was>1. 96, the local clusters were identified as high-value correlations (statistically significant hotspot, meaning the city had a high number of cases and was surrounded by other cities with high number of cases as well); and if Z-value was <−1.96, the local clusters can be identified as low-value correlation (statistically significant coldspot, meaning the city had a low number of cases). We conducted local spatial autocorrelation at city level to detect the hotspots and coldspots of HCV infection in mainland China.

## Results

### Basic information

**Figure 1:**
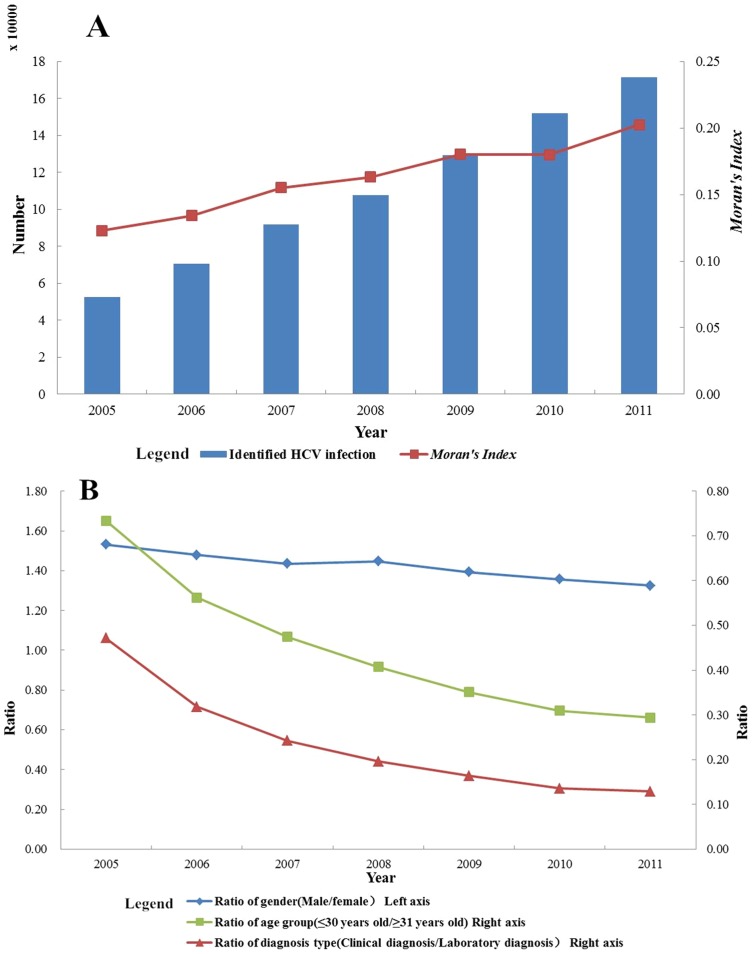
(A) Number and *Moran*'*s Index* of identified HCV infection cases by year. (B) Ratio of gender, age group and diagnosis type of identified HCV infection cases by year.

There were774,787 identified HCV infection cases that had accurate geographic information during the study period (2005–2011).7,568 cases(accounting for 0.98% of all identified) were excluded from the present study, due to lack of spatial information. Increased trend of number of identified HCV infection cases with years was observed (Figure 1A). During 2005–2011, the overall gender ratio (male/female) was 1.39; the mean age of all cases was 47.31 years (95%CI: 47.27 to 47.35) and, 16.51% of cases were ≤30 years. Almost 83.8%of HCV infection cases were identified by laboratory diagnosis and the rest were identified by clinical diagnosis.

### Trend analysis

The ratio of gender (Male/Female) was generally decreasing, 1.53, 1.47, 1.43, 1.44, 1.39 and 1.35 from 2005 to 2011 respectively, Z-value  = −18.53 (P<0.001). The ratio of age groups of cases (≤30 years old/≥31 years old) was 0.26 in 2005, while 0.16 in 2011. This trend also has statistical significance (Z-value  = −51.03, P<0.001).

Clinically diagnosis case is defined as a patient was detected by clinically agencies when he/she seek for treatment, while laboratory diagnosis case was simply detected by laboratory. The ratio of diagnosis type (Clinical diagnosis/Laboratory diagnosis) was also presented decreasing trend during study period, from 0.47 in 2005 to 0.12 in 2011 (Z-value  = −130.47, P<0.001). The trend analyses were described in Figure 1B.

### Spatial analysis

**Figure 2:**
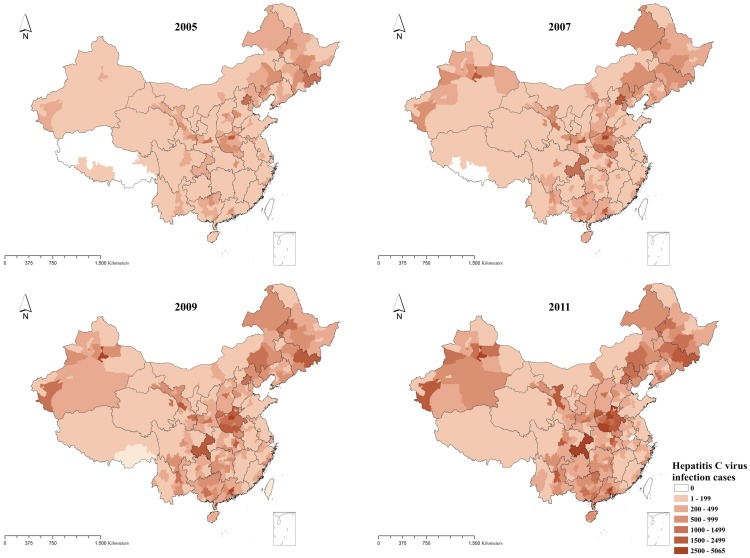
Geographical distribution of the number of identified HCV infection cases reported at city level in the years 2005, 2007, 2009 and 2011 in China.

The geographical distribution of identified HCV infection cases was found to be unbalanced in the mainland of China. As figure 2 presented, most cities have reported HCV infection cases during the study period, and the number of cities which have reported HCV infection cases were 339,341,341,342 in the year of 2005, 2007, 2009 and 2011, respectively. Henan province, Guangdong province, Guangxi Zhuang Autonomous Region, Xinjiang Uygur Autonomous Region and Jilin province have respectively reported more than 40,000 HCV infection cases during the period of 2005 to 2011. The total number of HCV identified cases in these five regions is 340,209, which accounted for 43.91% of all cases.

Firstly, general spatial autocorrelation was conducted for the cumulative number of HCV infection diagnosed between 2005 and 2011 by using distance matrix, and the value of *Moran's Index* was 0.178 and *P*<0.01. Then we conducted general spatial autocorrelation for the number of identified HCV infection cases of each year from 2005 to 2011(Table 1). The results indicated that HCV epidemic clustered in the mainland of China for total year and by each year, and the value of *Moran's Index* was increasing from 2005 to 2011 in general, meaning the strength of clustering of HCV cases got severer year by year (Figure 1A).

**Table 1:** Results of general spatial autocorrelation of from 2005 to 2011.

Year	*Moran's Index*	*Z*-value	*P*-value
2005	0.123	9.497	<0.01
2006	0.134	10.458	<0.01
2007	0.155	11.983	<0.01
2008	0.163	12.528	<0.01
2009	0.180	13.771	<0.01
2010	0.180	13.789	<0.01
2011	0.203	15.460	<0.01
Total(2005–2011)	0.178	13.655	<0.01

In order to figure out whether the stronger clusters resulted from the possible hotspots or coldspots and then to find them in specific area, local spatial autocorrelation was conducted by using the same matrix as general spatial autocorrelation.

**Figure 3:**
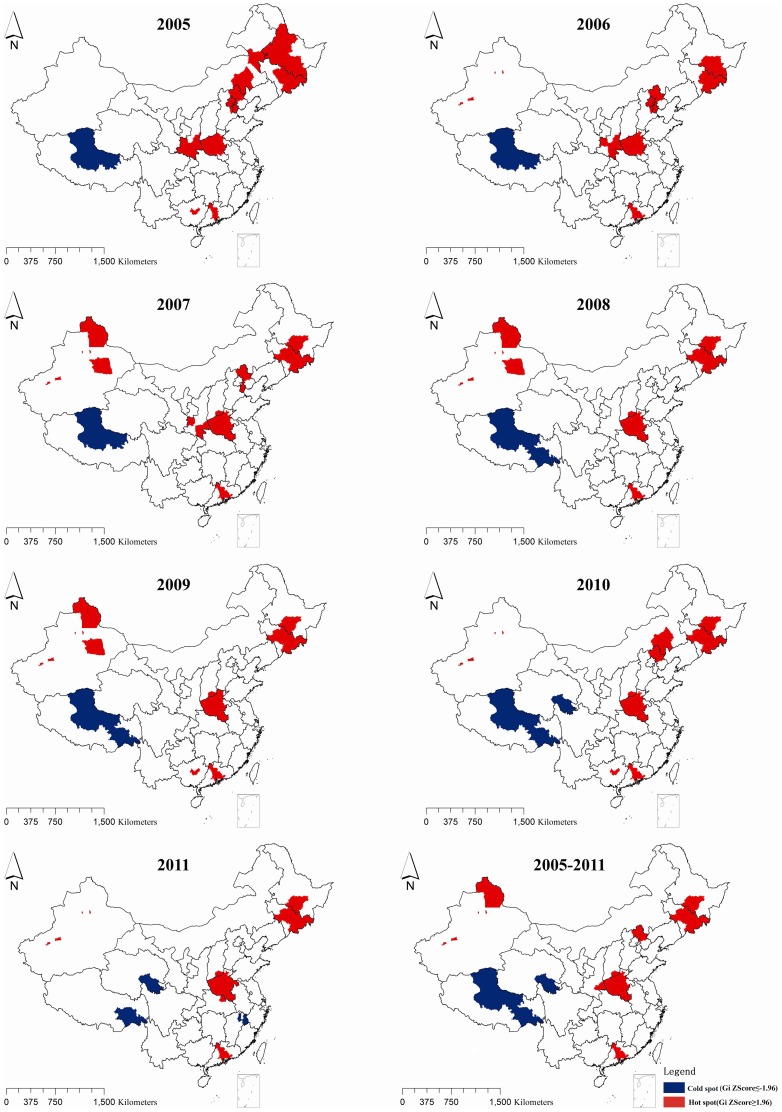
Hotspots and Coldspots of identified HCV infection cases at city level by year in China, 2005–2012.

As figure 3 showed, in general, hotspots concentrated in the areas where there was huge number of HCV infection cases, for example, Henan province and Jilin province etc. However, new hotspots were also observed in Hebei, Beijing, Tianjin and other provinces in recent years.

The coldspots were mainly limited to three areas: Tibet, Qinghai and Jiangxi. Tibet was always one of the coldspots in each year from the 2005 to 2011. During this period, the number of hot spots at city level decreased from 42 in 2005 to 31 in 2011, however, the number of coldspots increased slightly at the same period. The result of local spatial autocorrelation for the cumulative number of HCV infection cases reported between 2005 and 2011 was little different from the results of each year during this period. The details of hotpots and coldspots were listed in Table 2, and the reason for listing hotspots in provincial level is we mainly discussed cluster in this level.

**Table 2:** Distribution of Hotspots and Coldspots of HCV infection in China from 2005 to 2011.

	Hotspots		Coldspots	
Year	Number (city level)	Province, Municipality or Autonomous region where the hotspots located	Number (city level)	Province, Municipality or Autonomous region where the coldspots located
2005	42	Henan, Jilin, Guangdong, Shaanxi, Inner Mongolia, Hebei, Tianjin, Beijing, Heilongjiang	1	Tibet
2006	36	Henan, Jilin, Xinjiang, Guangdong, Shaanxi, Hebei, Tianjin, Beijing, Heilongjiang	1	Tibet
2007	37	Henan, Jilin, Xinjiang, Guangdong, Shaanxi, Shanxi, Hebei, Tianjin, Heilongjiang	1	Tibet
2008	31	Henan, Jilin, Xinjiang, Guangdong, Shanxi, Heilongjiang	2	Tibet
2009	33	Henan, Jilin, Xinjiang, Guangdong, Guangxi, Shanxi, Heilongjiang	2	Tibet
2010	32	Henan, Jilin, Xinjiang, Guangdong, Guangxi, Inner Mongolia, Shanxi, Heilongjiang, Hebei	3	Tibet, Qinghai
2011	31	Henan, Jilin, Xinjiang, Guangdong, Hubei, Shanxi, Heilongjiang	3	Tibet, Qinghai, Jiangxi
2005–2011	32	Henan, Jilin, Xinjiang, Guangdong, Shaanxi, Shanxi, Hebei, Heilongjiang	3	Tibet, Qinghai

## Discussion

Our study showed that not only the identified number of HCV cases in mainland China increased, but also the cases tended to more clustering, which means targeted interventions may be very helpful in China. After the implementation of the blood donation law (1998), and the law requiring mandatory screening for anti-HCV before blood donation (1993) [1], [4] in China, HCV infected patients might have more opportunities to get tested, which could possibly lead to the increased number of detection of HCV infection cases.

According to the previous study [18], the reported incidence of HCV infection in China was gradually increased during 1997 to 2011, especially since CISDCP was established in 2004. The upsurge was observed in both the prevalent number of HCV infection and its incidence indicating the emerging HCV infection epidemic in China.

Due to risky behaviors male conduct, for example, intravenous drug use and paid-blood/plasma donation, males are prone to get infected by HCV compared with female. However, the gender ratio (male/female) of HCV cases was decreasing in the present study, which was consistent with other studies [11], [19]. The equality of women's status, for example, more chances to involve into social connections and continuous improvement of mobility might be the reason. The ratio of age groups (≤30 years old/≥31 years old) and diagnosis types (Clinical diagnosis/Laboratory diagnosis) both showed a decreasing trend.

In the present study, the proportion of people aged more than 30 years old was increasing by year, so older people became more likely to get infected by HCV. Persistent HCV infection risk factors caused by the cumulative effect (the number and degrees of risk factors growing with the HCV-infected patient getting older) and more opportunities to assess to the clinical invasive treatment might explain this phenomenon.

Much more infection cases were diagnosed by laboratory, indicating that large-scaled improvement of laboratory testing capabilities has promoted early detection to explore more cases than ever, which also contributed to the growing number of identified number of HCV cases. Instead, some hospitals might not have adequate capabilities to detect infection.

The number of cities which reported HCV infection cases remained stable by year, with the number of infection cases increasing in the same period. However, spatial analysis indicated that the hotspots and coldspots existed, which indicated the HCV infection and epidemic was not randomly distributed.

Henan province, Guangdong province, Guangxi Zhuang Autonomous Region, Xinjiang Uygur Autonomous Region and Jilin province accounted for more than 40 percent of identified HCV infection cases in China during the study period. The illegal blood/plasma donation, intravenous drug use and poorer economy status etc. might be attributed to HCV infection epidemic in these provinces. The northern and central areas reported more infection cases than the rest of regions in China during the study period. There are 11 provinces, municipalities or autonomous regions once had the hotspots located from 2005 to 2011, and the number of hotspots is decreasing by year. The central and border areas were the regions where the hotspots frequently located.

Owing to the poorer economy status, Henan province suffered from paid-blood/plasma donation since 1990s [20]. Although various interventions (voluntary donation, etc.) and blood donation law had been conducted for many years, the huge gap between demand and supplement motivates the blood mostly through employer-organized blood collection. However, the donors may not have been true volunteers, as they may be forced by the employer in some degrees [1]. In addition, the poor economy and low level of health status facilitated HCV infection in this area during the period (2005–2011).

Hotspots were observed in Guangdong province, Guangxi Zhuang Autonomous Region, and Xinjiang Uygur Region in most of 7 years. These provinces located in the heroin trafficking route, which begin from “Golden Triangle” and then go to the southwestern or west provinces in China [15]. The circulation of drug logically brings out some risky behaviors of HCV infection [21], for example, the needles or cottons sharing, which were also the major reasons for HIV infection [22]. The northern hotspots frequently located in Changchun (Jilin province), Harbin (Heilongjiang province), both of two cities are the provincial capitals, which bring together many of medical resources and there were more likely to have clinical infection than other areas. In addition, Yanbian Korean Autonomous prefecture (Jilin province) was another location that a lot of case clustered, nationality and sharing a household with someone who had hepatitis C are contributed to the infection, according to a case-control study [23]. The other cities which did not show hotspots might result from many reasons, for example, the report numbers of these cities were lower and distributed randomly. However, this part of work will be taken in the further researches to understand.

Comparing with the changing status of hotspots, the coldspots were confined to three provinces in this study, Tibet, Qinghai, and Jiangxi. Tibet owned coldspots during 2005 and 2011, and this phenomenon might due to life style and religion of Tibet. Expect for these factors, the poor health system to have enough capacity to detect HCV cases might also be a reason for that.

The large sample size and the use of GIS system for analyzing the geological distribution, hotspots and coldspots of the epidemic, might be considered as the important strengths of this study, however, our study has some limitations like other case-record based studies,

Firstly, our data relied on CISDCP and it has possibility that some provinces may underreport the numbers of infection cases. For example, some cases may have not been identified yet. However, with the rapid development of CISDCP and surveillance system, the influence of this bias in our study is weakened. Second, the number of identified HCV infection cases might be influenced by the intensity of anti-HCV test.

Even with the limitations, this study still demonstrated that there was an upsurge of HCV epidemic in China, and the randomly spatial distribution has not been reported in recent years. Given the rising number of HCV infection case identified, the decreasing number of cities of hotspots and the increasing number of cities of coldspots indicated the specialized intervention strategies and prevention programs which targeting highly epidemic areas seemed to be urgently required in China. Studies exploring the reason of cluster and the correlation between increasing number of cases and cluster might be a good research area for further studies.
